# H_2_S events in the Peruvian oxygen minimum zone facilitate enhanced dissolved Fe concentrations

**DOI:** 10.1038/s41598-018-30580-w

**Published:** 2018-08-23

**Authors:** Christian Schlosser, Peter Streu, Martin Frank, Gaute Lavik, Peter L. Croot, Marcus Dengler, Eric P. Achterberg

**Affiliations:** 1 Marine Biogeochemie, Helmholtz-Zentrum für Ozeanforschung, GEOMAR, Kiel, Germany; 2 Max-Plank-Institut für Mikrobiologie, 28359 Bremen, Germany; 3 iCRAG (Irish Centre for Research in Applied Geoscience), Earth and Ocean Sciences, NUI Galway, Galway, Ireland

## Abstract

Dissolved iron (DFe) concentrations in oxygen minimum zones (OMZs) of Eastern Boundary Upwelling Systems are enhanced as a result of high supply rates from anoxic sediments. However, pronounced variations in DFe concentrations in anoxic coastal waters of the Peruvian OMZ indicate that there are factors in addition to dissolved oxygen concentrations (O_2_) that control Fe cycling. Our study demonstrates that sediment-derived reduced Fe (Fe(II)) forms the main DFe fraction in the anoxic/euxinic water column off Peru, which is responsible for DFe accumulations of up to 200 nmol L^−1^. Lowest DFe values were observed in anoxic shelf waters in the presence of nitrate and nitrite. This reflects oxidation of sediment-sourced Fe(II) associated with nitrate/nitrite reduction and subsequent removal as particulate Fe(III) oxyhydroxides. Unexpectedly, the highest DFe levels were observed in waters with elevated concentrations of hydrogen sulfide (up to 4 µmol L^−1^) and correspondingly depleted nitrate/nitrite concentrations (<0.18 µmol L^−1^). Under these conditions, Fe removal was reduced through stabilization of Fe(II) as aqueous iron sulfide (FeS_aqu_) which comprises complexes (e.g., FeSH^+^) and clusters (e.g., Fe_2_S_2_|4H_2_O). Sulfidic events on the Peruvian shelf consequently enhance Fe availability, and may increase in frequency in future due to projected expansion and intensification of OMZs.

## Introduction

Iron (Fe) forms an important micronutrient, controlling marine phytoplankton growth and nitrogen fixation in vast regions of the global ocean^1–3^. Shelf sediments are recognized as an important source of Fe to coastal waters and the open ocean^4–6^. In particular, Fe supplied from shelf sediments in oxygen minimum zones (OMZs) results in highly elevated dissolved Fe (DFe) concentrations in coastal waters depleted in oxygen (O_2_) to concentrations below 2 µmol L^−1 ^^7,8^, thereby providing a potential Fe source for offshore transport^9^. The strength of the Fe flux from shelf sediments is significantly enhanced in case sediments become euxinic (hydrogen sulfide (H_2_S) present)^10–12^.

Upwelling of nutrient-rich deep waters across the Peruvian shelf region results in extremely high primary productivity. The sediments and water column in this region are severely depleted in dissolved oxygen (O_2_) as a result of bacterial respiration of sinking organic matter^13^, augmented by a sluggish ventilation accounting for a reduced lateral and vertical flux of oxygen into the Peruvian shelf region^14,15^. The Peruvian sediment pore water concentrations of DFe, predominantly in the reduced form Fe(II), are in the 1–30 micromolar range and hence several orders of magnitude higher than in the overlying bottom waters^16^. The steep DFe concentration gradients and anoxic conditions of the bottom waters facilitate enhanced Fe fluxes out of the sediments reaching 10 to 866 µmol DFe m^−2^ d^−1 ^^8^, which result in bottom water DFe concentrations of 20–300 nmol L^−1 ^^7,17^, with most of the DFe being present in the reduced form Fe(II)^18–21^.

Iron(II) is formed in anoxic sediments by the dissimilatory reduction of Fe(III) oxides as part of a sequence of microbially mediated redox reactions^22,23^. The layer of Fe(II) enrichment in the pore waters of the sediments is located above that of elevated H_2_S levels. Below the Fe(II)-rich zone, H_2_S is formed by the dissimilatory reduction of sulfate (SO_4_^2−^) and reacts with dissolved Fe(II) to form a wide variety of aqueous Fe complexes ([FeSH]^+^, [Fe(SH)_2_]°, [Fe(SH)_3_]^−^, etc.) and clusters (e.g., Fe_n_S_n_^0^ | 4H_2_O (n = 2 or 4)) (summarized as FeS_aqu_)^24^ followed by amorphous particulate Fe(II) monosulfides (Fe(II)S) (e.g. mackinawite (FeS_m_))^25–27^. Aqueous Fe sulfide clusters are ligated directly to H_2_O and the structure of the cluster is similar to the basic structure of mackinawite^24^. Due to similar structural homologies found for aqueous zinc and copper sulfide clusters, it has been suggested that the structure of the cluster in solution determines the form of the initial particulate phase^28,29^. However, mackinawite formation is thermodynamically favored when the ion activity product of H_2_S and Fe(II) exceeds the thermodynamic stability product of FeS_m_ (*log K*_*sp*_ = −3.6)^26^. This condition is typically met in euxinic sediments (e.g. Fe(II) >30 µmol L^−1^; H_2_S >10 µmol L^−1 ^^30^), and represents the initial step of Fe pyrite (FeS_2_) formation^25,27,31^.

The formation of FeS_m_ is also reported to occur in anoxic coastal waters and has been observed in permanently euxinic bottom waters of the Framvaren fjord (Norway), where Fe(II) and H_2_S levels are in the millimolar range^32^. Because H_2_S concentrations in these waters are extremely high, Fe sulfide precipitates in the water column as framboidal pyrite^33^. It is, however, unclear whether favorable conditions for Fe sulfide precipitation also occur in the coastal OMZ regions off Peru. The Peruvian OMZ features extremely low O_2_ concentrations (<50 nmol kg^−1^)^34–37^, with anoxic waters prevailing at depths between 10 to 500 m, which in near shore regions are in direct contact with shelf sediments^28^. The positioning of the OMZ over euxinic shelf sediments facilitates benthic supply of H_2_S and Fe(II), which accumulates periodically in the overlying waters^7,38^. The transient presence of H_2_S in Peruvian coastal waters have mainly been observed during the austral summer season and their occurrences have recently been associated with stagnant flow on the shelf ^39^. H_2_S and DFe in the Peruvian OMZ waters have been reported to reach concentrations as high as 13 µmol L^−1^ and 300 nmol L^−17^, respectively, during the upwelling season in austral summer and the formation of Fe(II)S minerals resulting in DFe removal has been suggested^40^.

Here we present new data from three locations with different environmental settings on the Peruvian shelf regarding DFe, Fe(II), H_2_S, nitrate (NO_3_^−^), nitrite (NO_2_^−^) and ammonium (NH_4_^+^) (with the sum of all N species = DIN), O_2_, and dissolved inorganic phosphorous (DIP) expressed as P* (P* = DIP − DIN/16)^41^. We examine the mechanisms that control DFe in the Peruvian OMZ, under contrasting conditions of presence and absence of enhanced water column H_2_S concentrations.

## Results and Discussion

**Figure 1:**
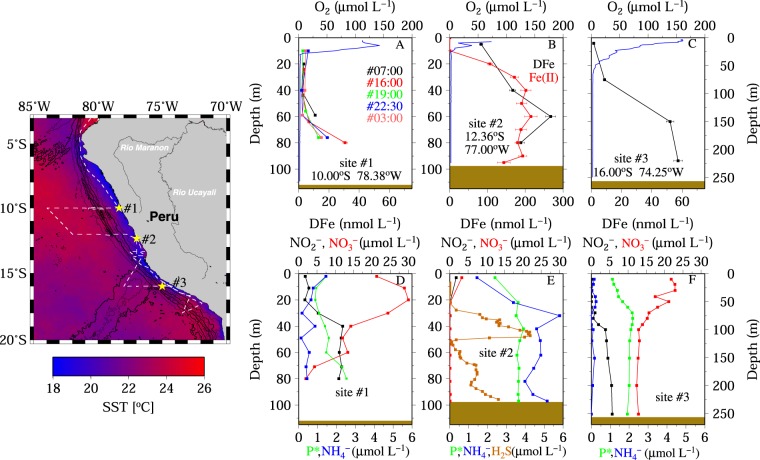
(Map) Sampling area of M77-3 (FS *Meteor*) in the Peruvian OMZ between December 2008 and January 2009. The cruise track is indicated by the dashed white line and the three Go-Flo sampling sites of this study are marked by the yellow stars and white station numbers. The colors show satellite sea surface temperature data (SST) in degree centigrade (MODIS). The GEBCO bathymetry is expressed by contour lines. The map was created by the open source software Generic Mapping Tool, GMT, Version 4 (http://gmt.soest.hawaii.edu). (**A**) Dissolved Fe concentration (DFe) in the water column at site 1 was sampled 5 times within 24 hours. O_2_ concentration is given by the blue line. (**D**) Nitrate (NO_3_ in red), nitrite (NO_2_ in black), Ammonia (NH_4_ in blue), and P* (in green) determined at site 1. Results from site 1 (**A** and **D**) and site 3 (**C** and **F**) are similarly assembled, but in B additionally the Fe(II) concentrations are shown in red and in E the water content of H_2_S is provided, a compound that was not detected at site 1 and 3.

Water column anoxia occurred at all three study sites during the upwelling season in December/January 2008/09 (Fig. 1A–C). The surface mixed layer extended down to ca. 10 m depth at site 1, 20 m at site 2 and 60 m depth at site 3, and was assessed using a fixed density difference criterion (Δσ = 0.125)^42^. At all sites, O_2_ concentrations decreased below the surface mixed layer to levels below the LOD of the CTD mounted O_2_ sensors. At site 2, O_2_ was 75 µmol L^−1^at 2 m depth, and decreased below 20 m to levels undetectable for the STOX sensor. The strong vertical oxygen gradient in the near-surface layers was a consequence of enhanced O_2_ consumption by microbial organic matter remineralization. At all three sites the anoxic conditions extended down to the seafloor.

The DFe levels in our study region were considerably higher than those reported for other coastal regions where OMZs prevail, e.g. 1.2–6.3 nmol L^−1^ on the north west African shelf ^4^. The profiles of DFe concentrations at sites 1 and 3 differed pronouncedly from site 2 (Fig. 1A–C). Dissolved Fe concentrations at site 1 increased from 2 nmol L^−1^ in the surface layer to 30 nmol L^−1^ at 80 m depth. Over a period of 24 h, five separately sampled casts indicated that DFe profiles remained essentially constant (Fig. 1A). Similar to site 1, DFe concentrations at site 3 increased from 2 nmol L^−1^ in the surface layer to 60 nmol L^−1^ at 90 m depth. Bottom water DFe values at both sites agreed well with reported values for anoxic Peruvian shelf bottom waters of 30 to 60 nmol L^−1 ^^17,19–21^. In contrast, DFe at site 2 increased from very high values of 80 nmol L^−1^ in the surface waters to ca. 200 nmol L^−1^ below 30 m depth, and remained relatively constant at ca. 200 nmol L^−1^ down to the seafloor (Fig. 1B). Below 30 m water depth, DFe occurred predominantly in the reduced Fe(II) form (Fig. 1B), an Fe species that has been analyzed just for site 2. Several studies with focus on Fe speciation have been already conducted in the Peruvian OMZ^9,20,21^. In agreement with our results, these studies showed that the reduced Fe(II) form represented the main DFe species in the anoxic part of the water column. However, Fe(II) concentrations in the water column determined during those studies were lower than found during our present study (Fe(II) = 15–73 nmol L^−1 ^^20^, 12–47 nmol L^−1 ^^19^, 0.2–16 nmol L^−1 ^^21^).

The water column at site 2 featured enhanced H_2_S concentrations, coinciding with high DFe levels (Fig. 1B,E). However, an increase in H_2_S concentrations at site 2 was observed with depth including a mid-depth maximum at 50 m (~4 µmol L^−1^; Fig. 1E) and ~3 µmol L^−1^ near the seafloor. Elevated DFe concentrations of up to 300 nmol L^−1^ during a H_2_S event reaching ~ 10 µmol H_2_S L^−1^ on the Peruvian shelf in 2012 have also been reported by Scholz, *et al*.^7^, with DFe and H_2_S being released by anoxic sediments.

**Figure 2:**
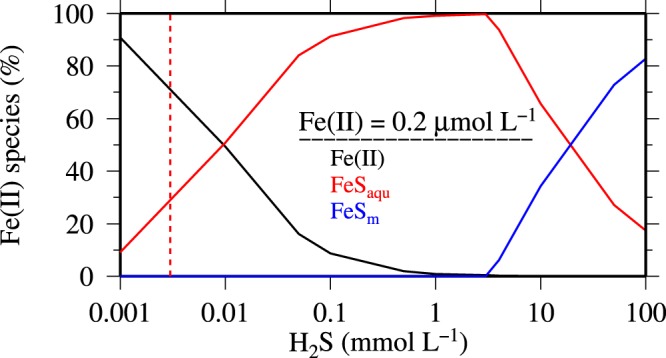
Distribution of Fe(II) species in percent (dissolved Fe(II) (black), soluble Fe (II) complexes/clusters (FeS_aqu_) (red), and particulate FeS_m_ (mackinawite) (blue)) at a constant Fe(II) concentration of 0.2 µmol L^−1^ and under increasing hydrogen sulfide (H_2_S) concentrations. The red dashed line represents the average H_2_S concentration in subsurface waters <50 m at site 2 in the Peruvian OMZ.

Removal of DFe through the formation of Fe(II) sulfide minerals has been reported for euxinic sediments^27^, deep-sea hydrothermal vent systems^43^, euxinic fjord waters^32^, and anoxic waters in the bottom boundary layer of the Peruvian OMZ^40^. Using Visual MINTEQ. 3.1^44^, we calculated the species distribution of dissolved Fe(II), aqueous sulfide (FeS_aqu_: *log K* = 5.62^24^), and Fe(II)S in the crystal structure of mackinawite (*log K* = −3.6^24^), an ubiquitous mineral in low temperature aqueous environments, to determine if Fe(II)S formation in the water column at site 2 was feasible (Fig. 2) (concentrations of model parameters used are listed in supplementary material S1). At 13 °C and pH 7.65, which is typical for subsurface waters on the Peruvian shelf^45,46^, and concentrations of Fe(II) of 200 nmol L^−1^ and H_2_S levels of 3 µmol L^−1^, which reflect the bottom water composition at site 2, the ion activity product of the educts (log *IAP* = − 5.466) is below the typical apparent solubility product of mackinawite of log *K’*_*sp*_ = − 3.6^44^ (undersaturation with respect to mackinawite: log *IAP* - log *K’*_*sp*_ <0). To validate the robustness of the model a sensitivity test was executed by increasing pH and temperature which would increase log *IAP* and enhance the likelihood of mackinawite formation. At 40 °C and pH 8.5, obviously very unrealistic conditions for the Peruvian OMZ, the log *IAP* increased slightly to −4.61. This suggests that under the transient conditions typically encountered, the formation of mackinawite is thermodynamically unfavorable in the anoxic bottom waters off Peru.

The mackinawite formation will only commence at Fe(II) and H_2_S concentrations in the higher micromolar range. Our modelling indicates that at pH 7.65, 13 °C and 200 nmol Fe(II) L^−1^ more than 2 mmol L^−1^ H_2_S are required to facilitate the formation of mackinawite (Fig. 2). Further, noticeably less H_2_S is required (~10 µmol L^−1^) at higher Fe(II) levels of >3 µmol L^−1^ to attain mackinawite saturation. These required enhanced H_2_S and Fe(II) concentrations for the formation of Fe(II) sulfide minerals in seawater are in agreement with other modelling studies (Fe ≥ 3 µmol L^−1^; H_2_S ≥ 50 µmol L^−1−^^47^) and earlier observations in anoxic regions such as the Framvaren fjord (Fe ≥ 2 µmol L^−1^; H_2_S ≥ 1 mmol L^−1^ ^32,48^), Black Sea (Fe ≥ 0.3 µmol L^−1^; H_2_S ≥ 30 µmol L^−1^ ^49,50^), and Baltic Sea (Fe ≥ 2.34 µmol L^−1^; H_2_S ≥ 52 µmol L^−1 ^^51^).

In H_2_S free seawater more than 75% of Fe(II) occurs as a truly dissolved free cation, while the remainder will form complexes with hydroxide (OH^−^), carbonate (CO_3_^2−^), and chloride (Cl^−^) ions^52^. In the presence of an excess of H_2_S over Fe(II) and above pH 7.5, FeS_aqu_ becomes the dominant Fe(II) species^24^ (Fig. 2). Formation of thiols in sediments^53^ may result in Fe(II)-thiol complexes, with thiols also facilitating reduction of Fe(III)^54^. Organic ligands complexing with Fe(III) serve a similar purpose in oxic waters to stabilize Fe^55^. In the sulfide containing Peruvian waters at site 2, the Fe distribution in the water column was therefore controlled by the total Fe flux from the sediments rather than the equilibrium concentrations of their solid phases. Prolonged sulfidic periods will therefore result in an increase in DFe concentrations in the anoxic water column, as formation of Fe(II)S precipitates like mackinawite is unfavorable at the Fe(II) and H_2_S concentrations so far observed in this region.

The formation of FeS_aqu_ stabilizes DFe via an increased soluble pool (as opposed to oxygenated waters in which DFe is dominated by Fe(III)-ligands and colloids^56^), and hence facilitates vertical diffusive DFe fluxes in the Peruvian OMZ. Vertical diffusive DFe fluxes were determined for site 2 by combining eddy diffusivities determined from microstructure measurements sampled in the same region during January 2012 (FS Meteor cruise M92^39^) and DFe concentration from this study. Altogether, 102 microstructure profiles were collected at shelf stations with bottom depth between 80 m and 100 m. The microstructure shear and temperature measurements were performed using a MSS90-D profiler (S/N 32, Sea & Sun Technology). Standard processing procedures were used to determine the rate of kinetic energy dissipation of turbulence in the water column (please see Schafstall *et al*.^57^ for a detailed description). Subsequently, eddy diffusivities were determined from ${K}_{\rho }={\rm{\Gamma }}\varepsilon {N}^{-2}$ where $N$ is stratification and ${\rm{\Gamma }}$ is mixing efficiency for which a value of 0.2 was used (Fig. S1)^58^. Between mid-depth waters (40 m) and the surface (10 m), an average eddy diffusivity of K_ρ_ = 3.1 × 10^−4^ m^2^ s^−1^ was obtained (Fig. S1). We employed the method of de Jong *et al*.^59^ and obtained a vertical diffusive DFe flux ${F}_{DFe}={K}_{\rho }\frac{\partial DFe}{\partial z}$ (with z being depth) from mid-depth waters at 40–60 m into the surface mixed layer of 101 µmol m^−2^ d^−1^. Upper and lower 95% confidence limits were determined to be 164 µmol m^−2^ d^−1^ and 69 µmol m^−2^ d^−1^ based on a Gaussian error propagation^57^. This vertical diffusive flux is in a range similar to reported benthic DFe fluxes (10–866 µmol m^−2^ d^−1^) from shallow Peruvian shelf sediments^8^. It indicates an important role of FeS_aqu_ in the anoxic waters of the Peruvian OMZ, which through stabilization facilitates Fe supply to surface ocean phytoplankton communities on the shelf and possibly further afield by filaments and mesoscale eddies that move off shore, away from the coast zone^60^.

In the absence of H_2_S in the anoxic water column at sites 1 and 3, DFe concentrations were 72 to 94% lower in bottom waters than at site 2. Vertical diffusive water column fluxes calculated using a K_ρ_ of 3.1 × 10^−4^ m^2^ s^−1^ at sites 1 and 3 were concurrently reduced by 88 to 97% (7.60 ± 4.13 µmol m^−2^ d^−1^), which indicates that supply and removal processes in anoxic water columns may differ and may depend on controls other than O_2_.

Organic matter remineralization processes in anoxic environments are mainly controlled by anaerobic microbial processes involving nitrogen, sulfur and Fe for electron-transfer reactions^61^. Of relevance for the Peruvian OMZ is the reduction of nitrate, coupled with the oxidation of H_2_S by filamentous bacteria of the family *Desulfobulbaceae*^62,63^. A similar coupling of anaerobic microbial denitrification with Fe(II) oxidation has been documented for chemolithotrophic organisms in freshwater sediments^64,65^, suboxic aquifers^66^, marine coastal sediments^67^, and for the anoxic water column off the coast of Peru, where Fe(II) oxidizers such as *Marinobacter aquaeolei* are active^7^.

Transient accumulations of H_2_S have been reported for anoxic Peruvian, Namibian and Indian shelf waters that were depleted in nitrate/nitrite^68–72^. These euxinic waters form the extreme end point of ocean conditions, with fully oxygenated waters being the opposite end point^73^. We suggest that the coupled process of anaerobic microbial denitrification and H_2_S oxidation depletes nitrate and allows DFe to accumulate in anoxic bottom waters in the Peruvian OMZ. The extent of anaerobic microbial denitrification in the Peruvian anoxic environments is mainly controlled by H_2_S levels and less by Fe(II) given that H_2_S concentrations are an order of magnitude higher than those of Fe(II).

**Figure 3:**
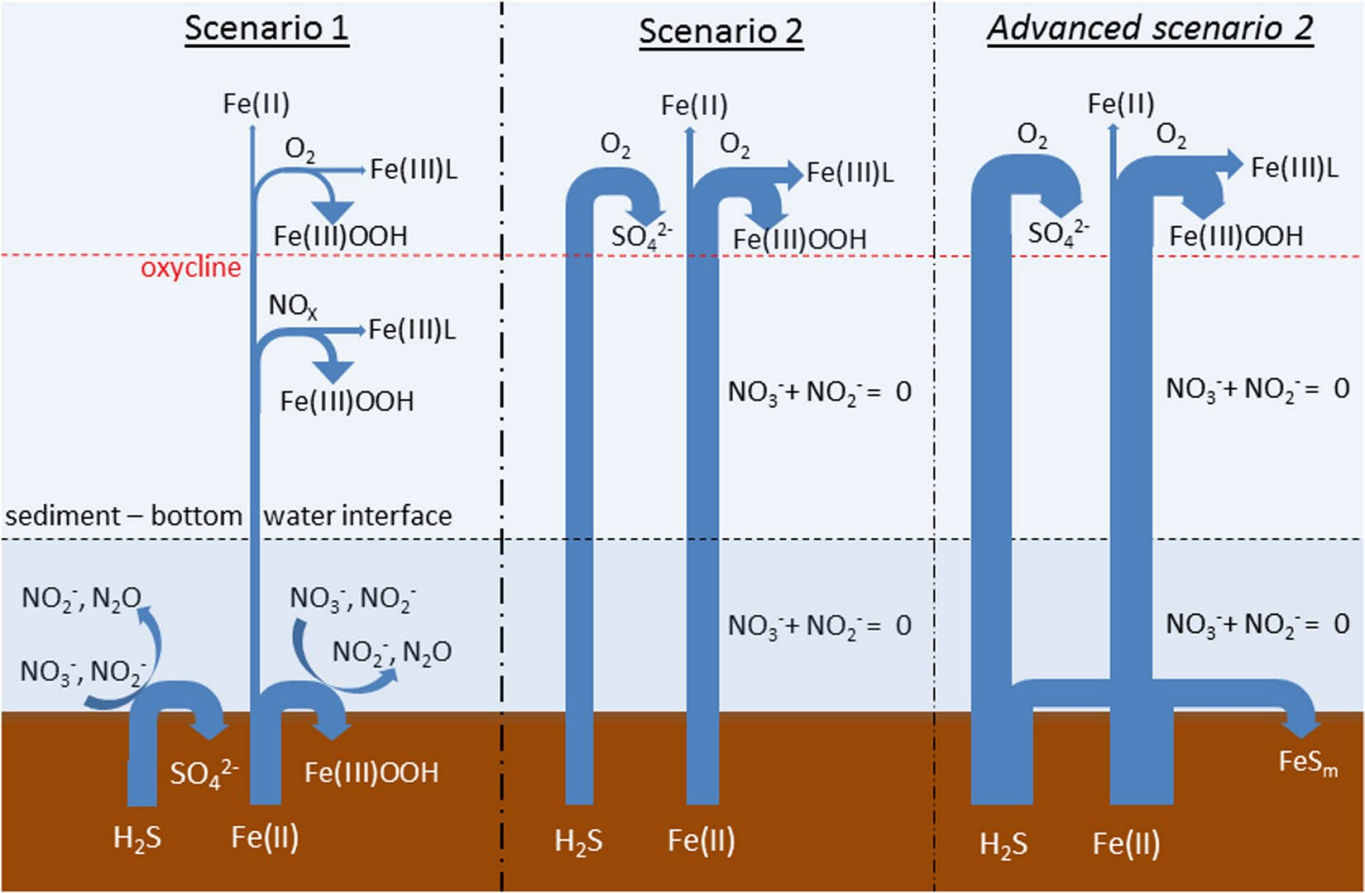
The left part of the sketch illustrates Scenario 1: H_2_S and the majority of Fe(II) are removed at the sediment-bottom water interface by oxidizing both compounds with NO_3_^−^ and NO_2_^−^ to sulfate (SO_4_^−^) and iron oxyhyroxides (Fe(III)OOH). In the water column, Fe(II) is oxidized by NO_3_^−^ and O_2_ with subsequent formation of particulate Fe(III)OOH and organically complexed Fe (Fe(III)L). A small fraction of Fe(II) is kept in solution by photochemical reduction processes in the surface. The middle part of the sketch illustrates Scenario 2: After H_2_S reduced most of the NO_3_^−^/NO_2_^−^ present in the sediments, H_2_S and Fe(II) can diffuse without restriction across the sediment-water interface and upwards in the water column until they are oxidized at the oxycline, with subsequent formation of particulate Fe(III)OOH, organically complexed Fe (Fe(III)L) and sulfate (SO_4_^−^). Similar to Scenario 1, a small fraction of Fe(II) is kept in solution by photochemical reduction processes in surface waters. The right part of the sketch illustrates Advanced scenario 2: Similar to scenario 2, but due to long lasting sedimentary release, H_2_S and Fe(II) concentrations increase in the water column. We hypothesize that elevated concentrations of H_2_S and Fe(II) in the millimolar range facilitate the precipitation of mackinawite (FeS_m_) at the sediment–bottom water interface.

Depending on H_2_S and nitrate concentrations, two distinct and one advanced scenarios can be envisaged (Fig. 3). Scenario 1: Under conditions of an anoxic water column (no measurable H_2_S) above the sediments and in the presence of nitrate and nitrite (i.e. denitrification and anammox has not fully removed nitrate and nitrite) bacteria at the sediment-water interface will reduce these nitrogen species and thereby utilize both H_2_S and Fe(II). During that process all the sulfide and a significant fraction of the Fe(II) will be oxidized. Insoluble Fe(III)oxyhydroxide particles are consequently formed near the sediment-bottom water interface, leading to an accumulation of reactive solid Fe phases near the sediment surface. Any Fe(II) that diffuses across the sediment-bottom water interface into the water column is then oxidized by O_2_/H_2_O_2_ or, in the absence of O_2_, via nitrate-dependent Fe(II) oxidizing microbes with the Fe remaining either as organically complexed Fe(III) (FeL), or is lost by scavenging processes and insoluble particle formation, as was documented by Heller *et al*.^18^. This situation was observed at sites 1 and 3 (Fig. 1A–C and F) and is illustrated in Fig. 3 (left schematic).

Scenario 2: Reductive removal of the nitrate and nitrite pool in the sediment, in association with H_2_S oxidation, allows enhanced sediment-water H_2_S and Fe(II) fluxes resulting in an accumulation of these species in the water column. The oxycline in the upper water serves as a removal filter, with e.g. oxidation of Fe(II) to Fe(III) in the presence of oxygen and hydrogen peroxide^74^. This scenario is proposed for site 2 (Fig. 1B and E) and resulted in the sulfidic event with H_2_S concentrations of up to 4 µM and Fe(II) concentrations of up to 200 nM (Fig. 3, middle schematic). We assume that such a scenario is temporarily confined. Advanced scenario 2: Long lasting release of sedimentary H_2_S and Fe(II) raises concentrations of both compounds in the water column, with highest concentrations expected at the sediment-bottom water interface. We hypothesize that under such conditions, log *IAP* > log *K’*_*sp*_, resulting in the formation of mackinawite at the sediment-bottom water interface (Fig. 3, right schematic). Due to a lack of observational data, we do not know if such an advanced scenario can occur in the more turbulent Peruvian OMZ, or if it just arises in water bodies as stagnant as the Black Sea^49^ and the Framvaren fjord^32^.

The coupling of Fe(II) and nitrite in OMZs is under debate in the scientific community. Hong and Kester^19^ found a linear relationship between nitrite and Fe(II) for samples from the OMZ along the Peruvian shelf and suggested this represented a common sediment source. More recently several trace metal studies conducted in open ocean OMZs observed a deep Fe(II) maximum that coincided with elevated nitrite and DFe (Fe(II) + Fe(III)) levels at the same depths. In the Peruvian OMZ a filament with elevated Fe(II) and nitrite concentrations in the center of the otherwise DFe rich OMZ has been located in the anoxic core zone between 300 m and 400 m water depth^9,20,75^. The filament extended from the coast to ca. 1,000 km off-shore and was related to off-shore Fe transport. A similar pattern has also been described for the OMZ in the Arabian Sea^76,77^ and both were interpreted as a coupling between nitrate reduction and Fe(II) accumulation, with Fe(III) being microbially reduced^78^. In contrast, Rickard and Luther III^24^, Scholz, *et al*.^7^, and Heller, *et al*.^18^ suggested a role for nitrite in the oxidation of Fe(II) and an accumulation of particulate Fe oxyhydroxides in the anoxic water column off Peru.

Periodic sulfidic events occur in the Peruvian OMZ. Our observations indicate that the present conditions are not favorable for the formation of mackinawite in the anoxic Peruvian waters given that Fe(II) and H_2_S concentrations remain consistently too low to exceed the solubility product of Fe(II) sulfide minerals. At micromolar H_2_S concentrations, aqueous Fe sulfide complexes and clusters become the dominant Fe(II) species and buffer DFe through prevention of scavenging, thereby enhancing Fe solubility in the euxinic water column.

Oxygen minimum zones are projected to expand and intensify as a consequence of reduced oxygen solubility related to ocean warming, increased stratification of the water column^15,79^, changes in oxygen consumption via biotic respiration, and changes in the large-scale overturning circulation^80^. This will likely result in more frequent H_2_S events and associated enhanced Fe(II) concentrations in coastal OMZs^72^. For the eastern tropical South Pacific off Peru it has been shown that eddies frequently forming at the coast can transport coastal waters far offshore within days and weeks^81^. This scenario may enhance the supply of DFe to local surface waters and potentially to the Fe depleted South Pacific gyre system^3,82,83^, with positive feedbacks for primary productivity and nitrogen fixation.

## Materials and Methods

### Sampling locations and sampling

Seawater samples for trace metal analysis were collected at three sites on the Peruvian shelf during the upwelling season in December and January 2008/09 (RV *Meteor* cruise M77-3). Sites were located at 10.00°S, 78.38°W (1), 12.33°S, 77.00°W (2) and 16.00°S, 74.24°W (3) (Fig. 1). The water depth was 112 m at site 1, 98 m at site 2 and 255 m at site 3. Samples for trace metal analysis were collected using trace metal clean Go-Flo bottles (General Oceanics) attached to a Kevlar wire. Go-Flo bottles were deployed once at sites 2 and 3, while at site 1 the Go-Flo bottles were deployed five times within 24 h. After recovery, bottles were transferred into a clean lab container. The seawater samples were filtered using cartridge filters (0.2 µm, Sartobran 300, Sartorius) and dispensed into acid cleaned 1 L low density polyethylene (LDPE, Nalgene) bottles. The samples were then acidified with quartz distilled hydrochloric acid to pH 1.8 (17.8 µmol H^+^ L^−1^). Unfiltered seawater samples for on-board Fe(II) analysis were dispensed into opaque acid cleaned 60 mL LDPE bottles under normal filtered air. The Fe(II) analysis was carried out immediately after sample collection. All sample handling was performed in a laminar flow hood.

### Sample analysis

Dissolved Fe concentrations were determined half a year later using graphite furnace atomic absorption spectrometry (Perkin Elmer, 4100 ZL) following Grasshoff *et al*.^84^. The blank and limit of detection (LOD) (three times the standard deviation of the blank measurement) for Fe concentrations were 0.104 nmol L^−1^ and 0.079 nmol L^−1^, respectively. The accuracy of the analytical procedure was evaluated by the analysis of certified seawater standard NASS-5 (National Research Council of Canada) and SAFe. Our Fe values agree well with the certified values for NASS 5 and the SAFe data (NASS 5: 26.3 ± 1.1 nmol kg^−1^ (certified: 25.7 ± 2.0 nmol kg^−1^);SAFe S: 0.112 ± 0.013 nmol kg^−1^ (census: 0.093 ± 0.008 nmol kg^−1^); SAFe D2: 0.83 ± 0.13 nM Fe (census: 0.933 ± 0.023 nmol kg^−1^)). The precision of the method is 3–5%.

Fe(II) concentrations were measured on samples collected at site 2 immediately upon collection by a chemiluminescence flow injection analysis following the method of Croot and Laan^85^, which has a LOD of 0.1 nmol L^−1^ Fe(II).

Nutrient and O_2_ samples were obtained using Niskin bottles (General Oceanics) on a stainless steel CTD rosette deployed at the same locations. Nutrient samples were analyzed for NO_3_^−^ and DIP using an autoanalyzer (TRAACS800, Bran&Lubbe) following Grasshoff *et al*.^86^. NO_2_^−^ was determined spectrophotometrically^86^ and NH_4_ was analyzed fluorometrically^87^ on board. Oxygen concentrations in the water column were measured by a Seabird O_2_ sensor that was calibrated with oxygen concentrations determined from CTD water samples (Winkler method^84^). The latter caused a LOD of 2 µmol kg^−1^. Additionally, at site 2 a switchable trace O_2_ sensor (STOX) with a LOD of 50 nmol kg^−1^ was used^36,37^. For H_2_S measurements seawater samples were collected with a pump-CTD^38^ and were analyzed spectrophotometrically^88^. At the pH of seawater HS^−^ represents the dominant hydrogen sulfide species, which is why the term H_2_S for our study refers to the sum of H_2_S, HS^−^, and S^2+^.

## Electronic supplementary material

Supplementary Material

## Data Availability

The dataset generated during this study is available in the GEOMAR-OSIS repository (https://portal.geomar.de/de/osis).
