# Supplementary material for: H2S events in the Peruvian oxygen minimum zone facilitate enhanced dissolved Fe concentrations
Source: Sci Rep. 2018 Aug 23;8:12642. doi: 10.1038/s41598-018-30580-w (PMC6107642; doi:10.1038/s41598-018-30580-w)
Supplement: Supplementary file 1 — Supplementary Material [file 41598_2018_30580_MOESM1_ESM.docx]

**H_2_S events in the Peruvian oxygen minimum zone facilitate enhanced dissolved Fe concentrations**

Christian Schlosser^1*^, Peter Streu^1^, Martin Frank^1^, Gaute Lavik^2^, Peter L. Croot^1,3^, Marcus Dengler^1^ and Eric P. Achterberg^1^

^1^Marine Biogeochemie, Helmholtz-Zentrum für Ozeanforschung, GEOMAR, Kiel, Germany

^2^Max-Plank-Institut für Mikrobiologie, 28359 Bremen, Germany

^3^iCRAG (Irish Centre for Research in Applied Geoscience), Earth and Ocean Sciences, NUI Galway, Galway, Ireland

S1 – Applied concentrations and stability coefficient of model parameters for Visual MINTEQ 3.1

| components | concentrations | |
| --- | --- | --- |
|  |  | |
| Na^1+^ | 481 mM | |
| K^1+^ | 10 mM | |
| Mg^2+^ | 54 mM | |
| Ca^2+^ | 11 mM | |
| Sr^2+^ | 90 µM | |
| Cl^-1^ | 650 mM | |
| Br^-1^ | 870 µM | |
| CO_3_^-2^ | 2 mM | |
| B(III) | 430 µM | |
| F^-1^ | 70 µM | |
| H_2_S (variable) | 0.001 – 100 mM | |
| Fe(II) | 0.2 µM | |
| pH | 7.65^1^ | |
|  |  | |
| included species | | |
| mackinawite | | log K_sp_ = -3.6^2^ |
| FeS_aqu_ | | log K = 5.62^3^ |
|  | |  |

Supplementary Figures


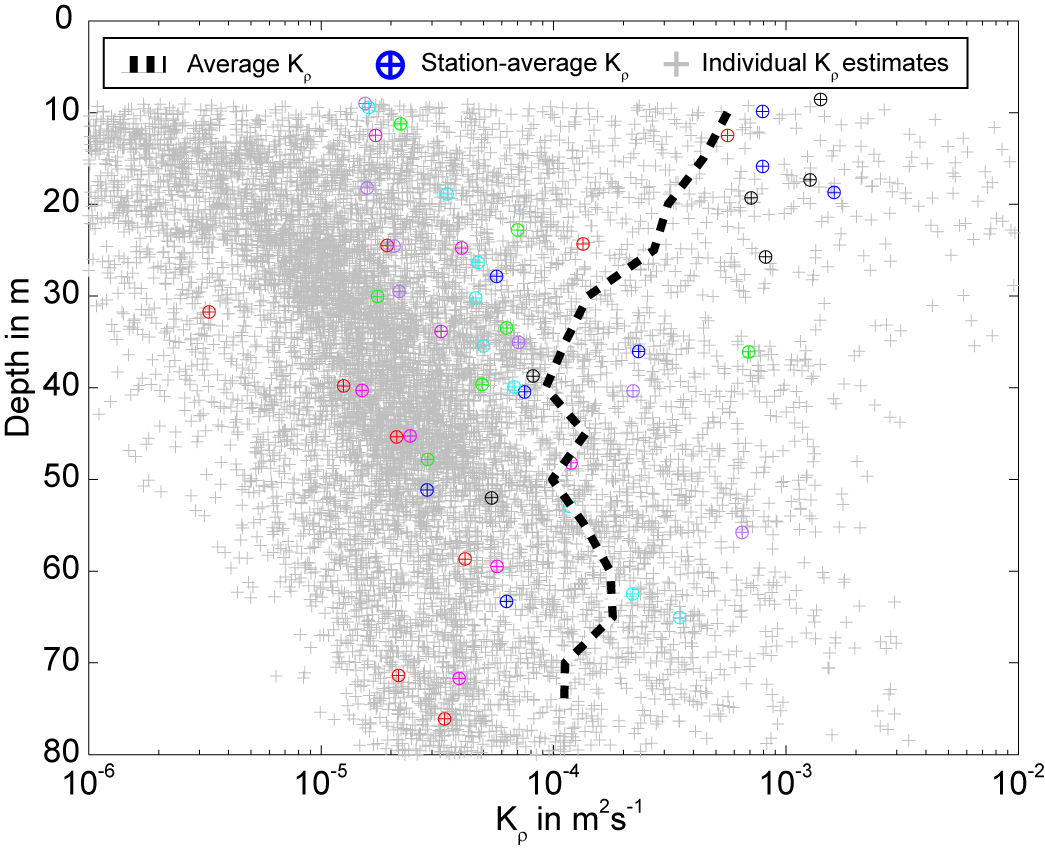


Figure S1: Shows vertical diffusivity (K_ρ_) versus depth. K_ρ_ was estimated from 102 microstructure deployments at stations with water depth between 80 and 100 m as described in Schaftstall et al. ^4^. The data was collected during FS Metor cruise M92-2 in January 2012 (for more detail please read Sommer et al. ^5^). Cruise M92-2 took place in the same OMZ region than Meteor cruise M77-3. The dashed black line indicates the average K_ρ_ at depth. The vertical diffusive DFe flux was calculated using an average value of K_ρ_ = 3.1 x 10^-4^ m^2^ s^-1^, derived from K_ρ_ values between mid-depth waters (40m) and the surface (10m).

References

1 Feely, R. A., Sabine, C. L., Hernandez-Ayon, J. M., Ianson, D. & Hales, B. Evidence for upwelling of corrosive “acidified” water onto the continental shelf. *Science* **320**, 1490-1492 (2008).

2 Gustafsson, J. P. *Visual MINTEQ ver. 3.1*, <<https://vminteq.lwr.kth.se>> (2014).

3 Rickard, D. & Luther III, G. W. Chemistry of Iron sulfids. *Chem. Rev.* **107**, 514-562 (2007).

4 Schafstall, J., Dengler, M., Brandt, P. & Bange, H. Tidal-induced mixing and diapycnal nutrient fluxes in the Mauritanian upwelling region. *J. Geophys. Res.: Oceans* **115**, 1-19 (2010).

5 Sommer, S., Dengler, M. & Treude, T. Benthic element cycling, fluxes and transport of solutes across the benthic boundary layer in the Peruvian oxygen minimum zone, (SFB754) – Cruise No. M92 – January 05 – February 03, 2013 – Callao (Peru) – Callao (Peru), Meteor-Berichte, M92, DFG-Senatskommission für Ozeanographie, 55 p. (2014).
